# Progress in Bacteriology

**Published:** 1902-05-10

**Authors:** 


### PROGRESS IN BACTERIOLOGY.

(*Continued from page* 85.)

**Diphtheria.**—Gorham[4] has contributed a very valuable review of Wesbrook's work on the morphological varieties of the Bacillus diphtheriæ. A plate illustrating the various forms of the bacillus is given, and to fully appreciate the contribution it is essential that the plate be closely studied. He divides diphtheria bacilli into three main classes: granular and barred types, such as are generally associated with clinical symptoms; and solid types, which are most frequent in convalescence from diphtheria, or which are found in the noses and throats of contacts or healthy persons. Briefly summarised, the conclusions of these two observers are as follow:—Granular types are predominant at the outset of the disease, but before the termination of the case these give place wholly or in part to barred and solid types. Rarely at the termination of a case granular types may be found to replace solid types. Diphtheria-like bacilli are more common in the nose than in the throat, and occur frequently in apparently healthy persons. The change from granular or barred types to solid forms is brought about by the influence of body fluids of persons who are becoming immune. The so-called xerosis, pseudo-diphtheria, or Hoffman's bacillus, are morphological varieties of the Bacillus diphtheriæ, which, although they may be capable of exciting clinical diphtheria, are seldom pathogenic to guinea-pigs. It seems probable that solid-staining types may regain their virulence. Neisser's stain is of no value in differentiating the barred or solid types of Bacillus diphtheriæ.

**Rheumatoid Arthritis.**—Poynton and Payne[5] described a diplococcus isolated after death from the knee joint of a man suffering from the osteo-arthritic variety of rheumatoid arthritis. Cultures were made on blood-agar, and when injected intravenously into rabbits gave rise to a non-suppurative arthritis associated with wasting of the neighbouring muscles. There was ulceration of the cartilages and lipping of the edges of the bones. These results were different from those obtained by the intravenous injection of the diplococcus rheumaticus described by the same workers, and no cardiac lesions were found. The diplococcus was demonstrated in sections of the cartilages from the case recorded.

**Bacteria in the Breath.**—From an examination of the lungs of healthy animals, Boni[6] concludes that these organs are not generally sterile, but contain a few micro-organisms, the pneumococcus predominating. Whilst he found the lungs of animals in the laboratory almost always sterile, the organs of freshly-killed beasts in slaughter-houses generally contained bacilli. Hubener recently showed that in ordinary speech bacilli are expelled from the mouth in little bubbles of saliva. Agar plates of a total surface of 200 s.cm. were placed at a distance of 50 cm. from a man, who was made to count aloud for 10 minutes. About

1,500 colonies developed on the plates after incubation.

**Chorea.** — Fornaca[7] recommends that in this disease the cerebro-spinal fluid should be systematically examined for micro-organisms. One-third of all cases of chorea follow infective diseases. Thus in a case of chorea following erysipelas he found streptococci not only in the cerebro-spinal fluid, but also in the blood and urine. In two other cases the cerebro-spinal fluid showed staphylococci.

**Ante-mortem Thrombi.** — Bryant[8] has made a bacteriological examination of the clot in three cases. In the first, a case of mitral stenosis, strepto- and staphylococci were present; in the second, a case of tubercular peritonitis with saphenous vein thrombosis, the proteus vulgaris was detected; and in the last, a case of carcinoma of the stomach in which the saphenous veins of both sides were blocked, the B. subtilis was present. Andrews[9] reports a case of emboli in the brachial and femoral arteries occurring in the course of a fatal case of malignant endocarditis due to the Bacillus coli communis.

**Micro-organisms and Granulation Tissue.** — Jürgelunas[10] finds that aseptic granulation tissue has a marked action on bacteria. The cells are bactericidal and phagocytic, and if the animal be immune to the particular bacillus then this perishes in the granulation tissue and is absorbed. The natural bactericidal action of granulation tissue is not affected by the nature of the dressing applied to the wound.

[4] Jour. of Med. Research, July, 1901. [5] Brit. Med. Jour., Jan. 11, 1902. [6] Deut. Arch. f. Klin. Med. Bd. LXIX. H 5-6. [7] Rep. Med., No. 74, 1901. [8] Lancet, May 11, 1901. [9] Lancet, Nov. 25, 1901. [10] Zieg. Beit. Bd. 29, H 1, 1901.

